# Spatial Habitat Features Derived from Multiparametric Magnetic Resonance Imaging Data Are Associated with Molecular Subtype and 12-Month Survival Status in Glioblastoma Multiforme

**DOI:** 10.1371/journal.pone.0136557

**Published:** 2015-09-14

**Authors:** Joonsang Lee, Shivali Narang, Juan Martinez, Ganesh Rao, Arvind Rao

**Affiliations:** 1 Department of Bioinformatics and Computational Biology, The University of Texas MD Anderson Cancer Center, Houston, Texas, United States of America; 2 Department of Neurosurgery, The University of Texas MD Anderson Cancer Center, Houston, Texas, United States of America; Beijing Tiantan Hospital, Capital Medical University, CHINA

## Abstract

One of the most common and aggressive malignant brain tumors is Glioblastoma multiforme. Despite the multimodality treatment such as radiation therapy and chemotherapy (temozolomide: TMZ), the median survival rate of glioblastoma patient is less than 15 months. In this study, we investigated the association between measures of spatial diversity derived from spatial point pattern analysis of multiparametric magnetic resonance imaging (MRI) data with molecular status as well as 12-month survival in glioblastoma. We obtained 27 measures of spatial proximity (diversity) via spatial point pattern analysis of multiparametric T1 post-contrast and T2 fluid-attenuated inversion recovery MRI data. These measures were used to predict 12-month survival status (≤12 or >12 months) in 74 glioblastoma patients. Kaplan-Meier with receiver operating characteristic analyses was used to assess the relationship between derived spatial features and 12-month survival status as well as molecular subtype status in patients with glioblastoma. Kaplan-Meier survival analysis revealed that 14 spatial features were capable of stratifying overall survival in a statistically significant manner. For prediction of 12-month survival status based on these diversity indices, sensitivity and specificity were 0.86 and 0.64, respectively. The area under the receiver operating characteristic curve and the accuracy were 0.76 and 0.75, respectively. For prediction of molecular subtype status, proneural subtype shows highest accuracy of 0.93 among all molecular subtypes based on receiver operating characteristic analysis. We find that measures of spatial diversity from point pattern analysis of intensity habitats from T1 post-contrast and T2 fluid-attenuated inversion recovery images are associated with both tumor subtype status and 12-month survival status and may therefore be useful indicators of patient prognosis, in addition to providing potential guidance for molecularly-targeted therapies in Glioblastoma multiforme.

## Introduction

Glioblastoma multiforme (GBM) is one of the most common and malignant brain tumors in humans. The general treatment of GBM generally involves surgical resection followed by combination of radiation therapy and chemotherapy (temozolomide). Even with multimodality treatment, the median survival rate of GBM patients is between 12 ~ 15 months [1].

Analysis of tumor heterogeneity has the potential to aid treatment of GBM, as different tumor subpopulations may respond differently [2–4]. It has been suggested that heterogeneity in gray-level intensity in magnetic resonance imaging (MRI) may be indicative of such distinct tumor habitats [5]. Such heterogeneity is often measured by texture analysis, which provides information about the spatial arrangement of gray-level intensities and quantifies the local characteristic patterns in an image. Texture analyses using modalities such as MRI, positron emission tomography (PET), and computed tomography (CT) have been shown to be promising for cancer prognosis [6–9].

Aside from texture analysis, recently, spatial heterogeneity of tumor with distinctly different MRI intensity characteristics was investigated using the concept of radiologically defined tumor regions (habitats) within the tumor [3]. Each of these regions or habitats can characterize regional variations in blood flow, cell density, and necrosis within the tumor, based on multiparametric measurements. In this study, we extended this investigation by treating such multiparametric tumor habitats as “multitype spatial point patterns,” a commonly used paradigm in ecological and spatial statistics. Apart from the abundance of these habitats, the spatial extents and proximity of these habitats might have physiologic and clinical relevance for assessment of treatment response.

Spatial point pattern analysis is the study of the spatial arrangements of points that describe the geometric structure and relationship of patterns in two-dimensional space [10]. Spatial point pattern analysis produces several diversity and proximity measurements such as the Simpson index and the Shannon index, which are quantitative measures of abundance of point types and their relative spatial proximities/relationships within a spatial region. Diversity analysis of spatial point patterns has been applied to understand spatial relationships among point patterns in ecology, astronomy and material science [11, 12]. To the best of our knowledge, this is the first study to use point process theory to study the spatial relationships of tumor habitats and assess their relationship with molecular status and clinical outcome. We sought to determine the association between the spatial habitat features derived from point patterns of tumor habitats and overall survival (OS) in GBM. Our results show that spatial habitat features derived from point patterns of MR tumor habitats are associated with 12-month OS status as well as molecular subtype status in GBM patients.

## Materials and Methods

### Data

We identified 74 GBM patients from The Cancer Genome Atlas on the basis of available post-contrast T1-weighted and T2-weighted Fluid Attenuated Inversion Recovery (FLAIR) image data from The Cancer Imaging Archive (TCIA): https://public.cancerimagingarchive.net/ncia/login.jsf as well as companion genomic information (Glioblastoma Multiforme TCGA, Provisional). Molecular information (describing gene expression—based molecular classification of GBM such as classical, mesenchymal, neural, and proneural subtype) [13] and survival data were obtained from the cBioPortal for Cancer Genomics (http://www.cbioportal.org). The patients’ TCGA IDs are listed in Table A in S1 Appendix and the information about frequent gene mutations in all the patients in this study is listed in Table B in S1 Appendix. Patients were assigned to one of two survival classes on the basis of their OS: ≤ 12 months or > 12 months. Thus, 31 patients had an OS of 12 months or less, and 43 patients had an OS of more than 12 months. This 12-month OS cut-off was based on the typical median survival time (12~15 months) in GBM, also used in other studies [14, 15]. The descriptive statistics regarding patient demographics are summarized in Table 1.

**Table 1:** Patient demographics.

Characteristics	OS ≤12 mo.	OS >12 mo.	Total
Male (n)	19	30	49
Female (n)	12	13	25
Age (y)	61.6 ± 15.3	54.5 ± 15.4	57.5 ± 15.7
Overall survival (mo.)	5.9 ± 3.0	24.8 ± 12.7	16.9 ± 13.6

The data for age and overall survival are means with standard deviations; OS—Overall survival

### Image Preprocessing

**Fig 1:**
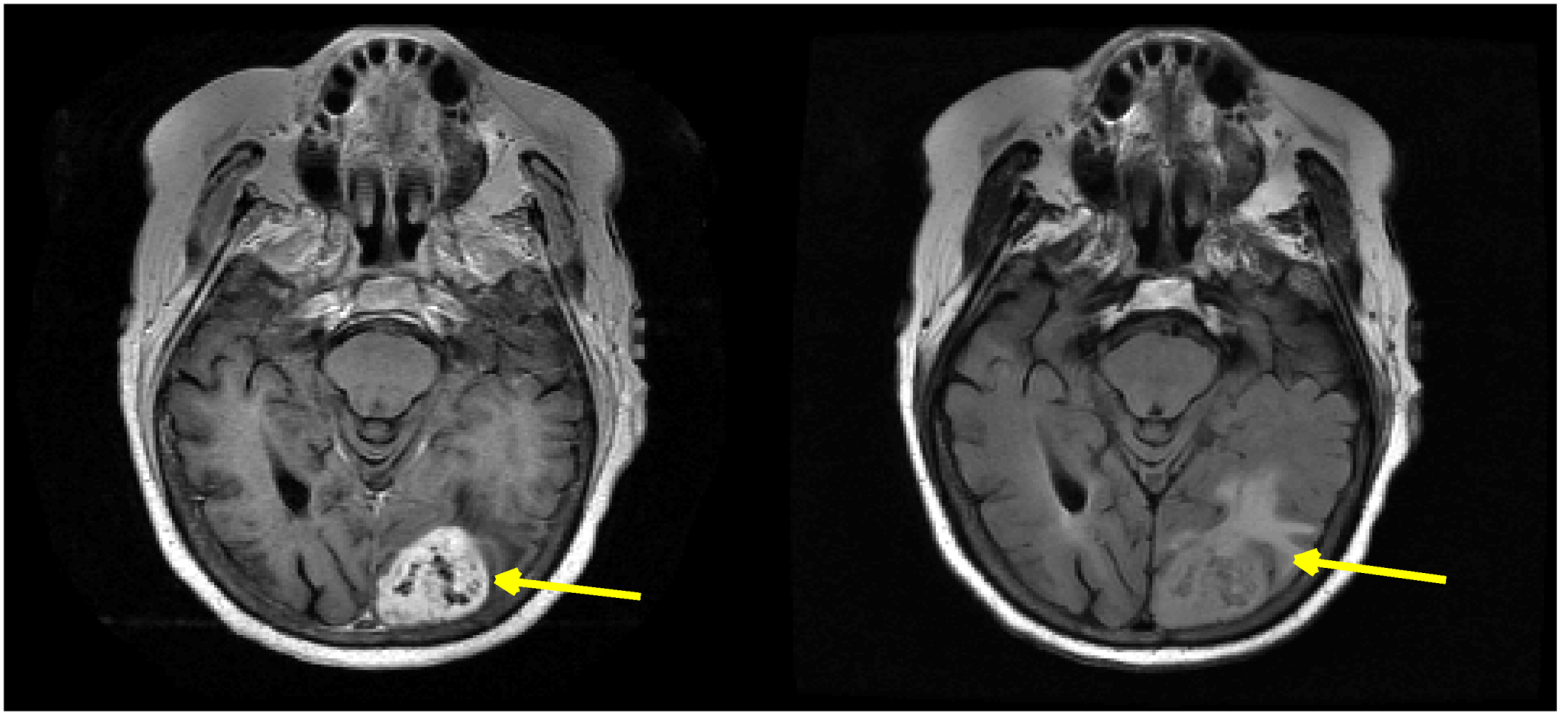
T1 post-contrast image (left) and T2 FLAIR image (right) after preprocessing. Arrows indicate the enhanced tumor area.

Image preprocessing procedures such as registration, non-uniformity intensity correction, reslicing, and intensity scaling were performed prior to multiparametric image analysis. First, 3-dimensional rigid registration was performed by spatially registering the T2 FLAIR image to the T1 post-contrast image as the reference image using Medical Image Processing, Analysis, and Visualization (MIPAV) software [16]. Subsequently, nonparametric nonuniform intensity normalization (N3) correction in MIPAV [17] was performed to correct MRI artifacts such as radiofrequency field nonuniformity, main magnetic field (B_0_) nonuniformity, and shading artifacts in MRI. Image reslicing was performed using the NIFTI toolbox in MATLAB v8.0 (The MathWorks Inc., Natick, MA) to obtain isotropic pixel resolution with 1 mm pixel size. Image intensities are quantized to 256 gray levels (Zhou et al., 2014) [3]. Fig 1 shows a T1 post-contrast image and a T2 FLAIR image after preprocessing.

### Identification of Tumor habitats

A tumor region of interest (ROI) was segmented semi-automatically by experts using the Medical Image Interaction Toolkit (www.mitk.org). The maximum tumor area slice in a T1 post-contrast image and the corresponding slice in a T2 FLAIR image were selected for this study.

**Fig 2:**
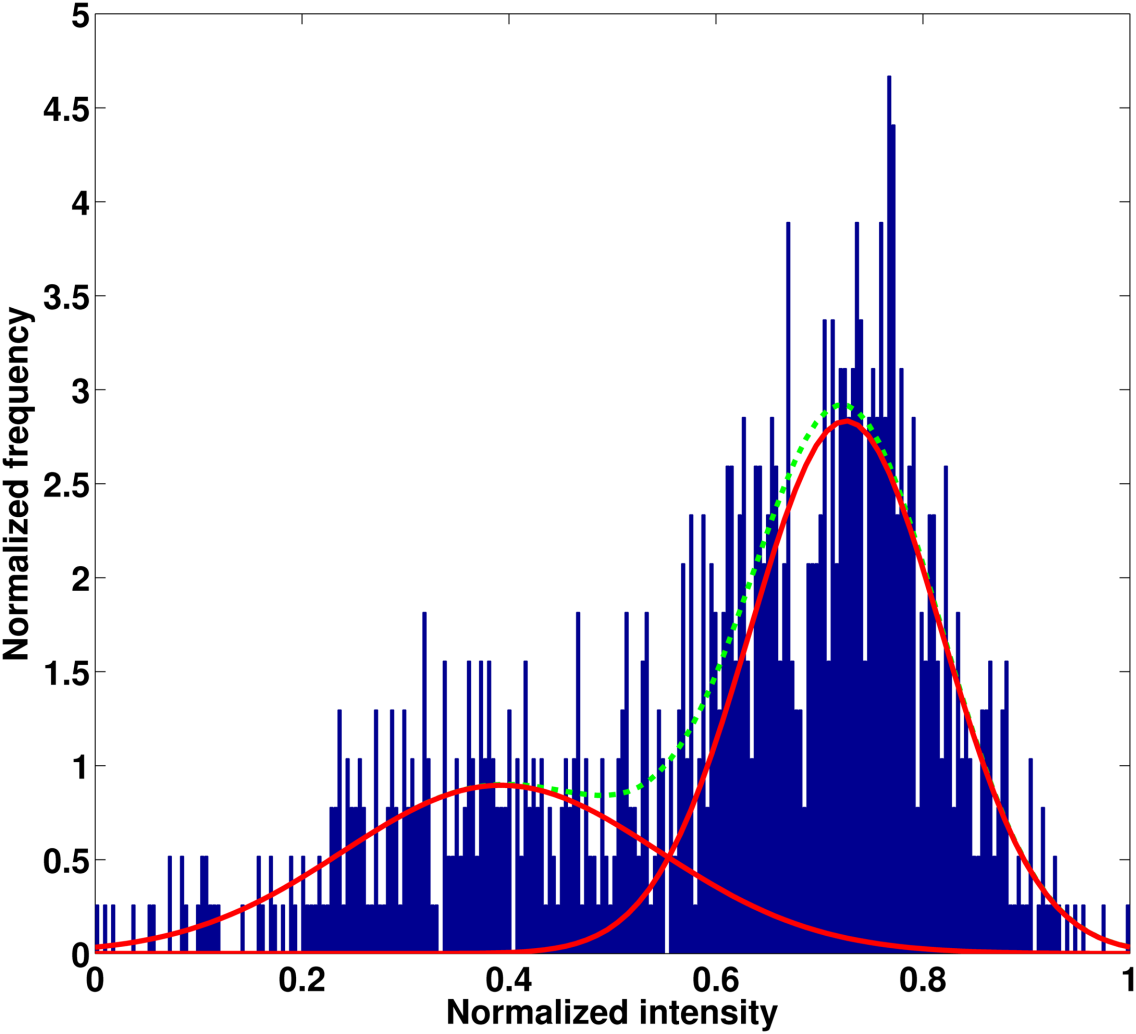
A representative 2D histogram of the ROI of a T1 post-contrast image. Intensity values are binned into two groups based on pixel membership to a Gaussian mixture model. Solid red line represents each Gaussian population; dotted green line represents total intensity distribution of the ROI.

Following intensity scaling of the segmented tumor region [3], the ROI was divided into two pixel groups based on its constituent pixel intensities using Gaussian mixture modeling [18, 19]. The intensities are assumed to follow a mixture of two Gaussian distributions, following previous work (Zhou et al., 2014) [3]. A representative intensity histogram for a T1 post-contrast signal is shown in Fig 2. Similar grouping of pixels was done with the linearly scaled T2 FLAIR signal. Each pixel within an ROI in a T1 post contrast image or T2 FLAIR image was assigned to a low- or high-intensity group (“habitat”) based on the cluster membership of that pixel. The threshold between the two Gaussian groups was determined by calculating the average of the means of the two Gaussian populations underlying the low-intensity pixel group and high-intensity pixel group within a tumor.

### Coordinates of the centroids

**Fig 3:**
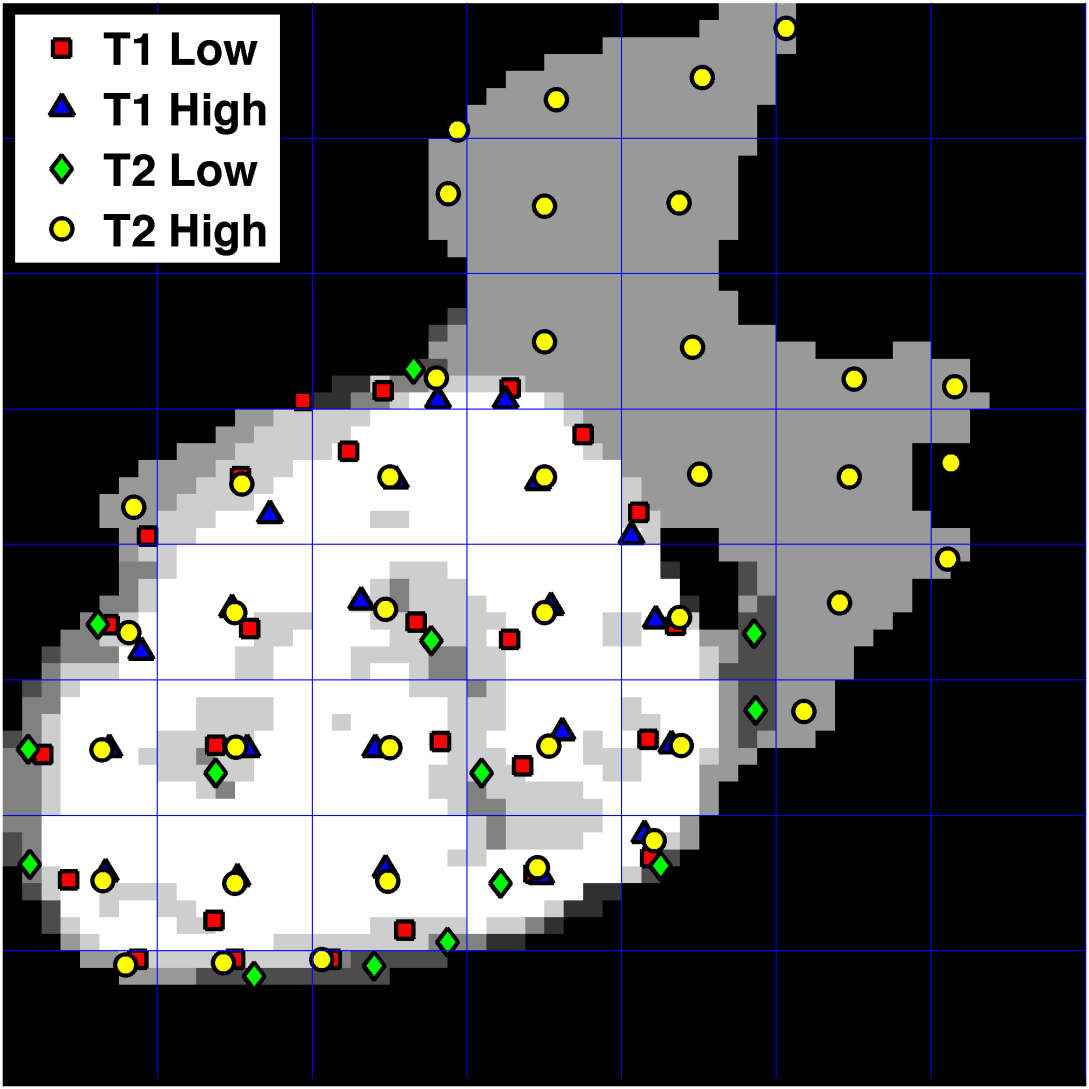
A sample ROI spatial map. A spatial map was combined with the low-intensity T1 post-contrast ROI, high-intensity T1 post-contrast ROI, low-intensity T2 FLAIR ROI, and high-intensity T2 FLAIR ROI. Each binary mask is equally divided into several bounding boxes and the size of each bounding box is 8×8 pixels. The coordinates of each centroid indicating the center of mass of each group inside of the small bounding grid box were calculated, and coordinates from all four regions were combined into a spatial map. The centroids of each subpopulation are designated by four different shapes based on group to illustrate the spatial relations between the four groups. Different gray levels of ROI represent each habitat and overlaps of habitats.

**Fig 4:**
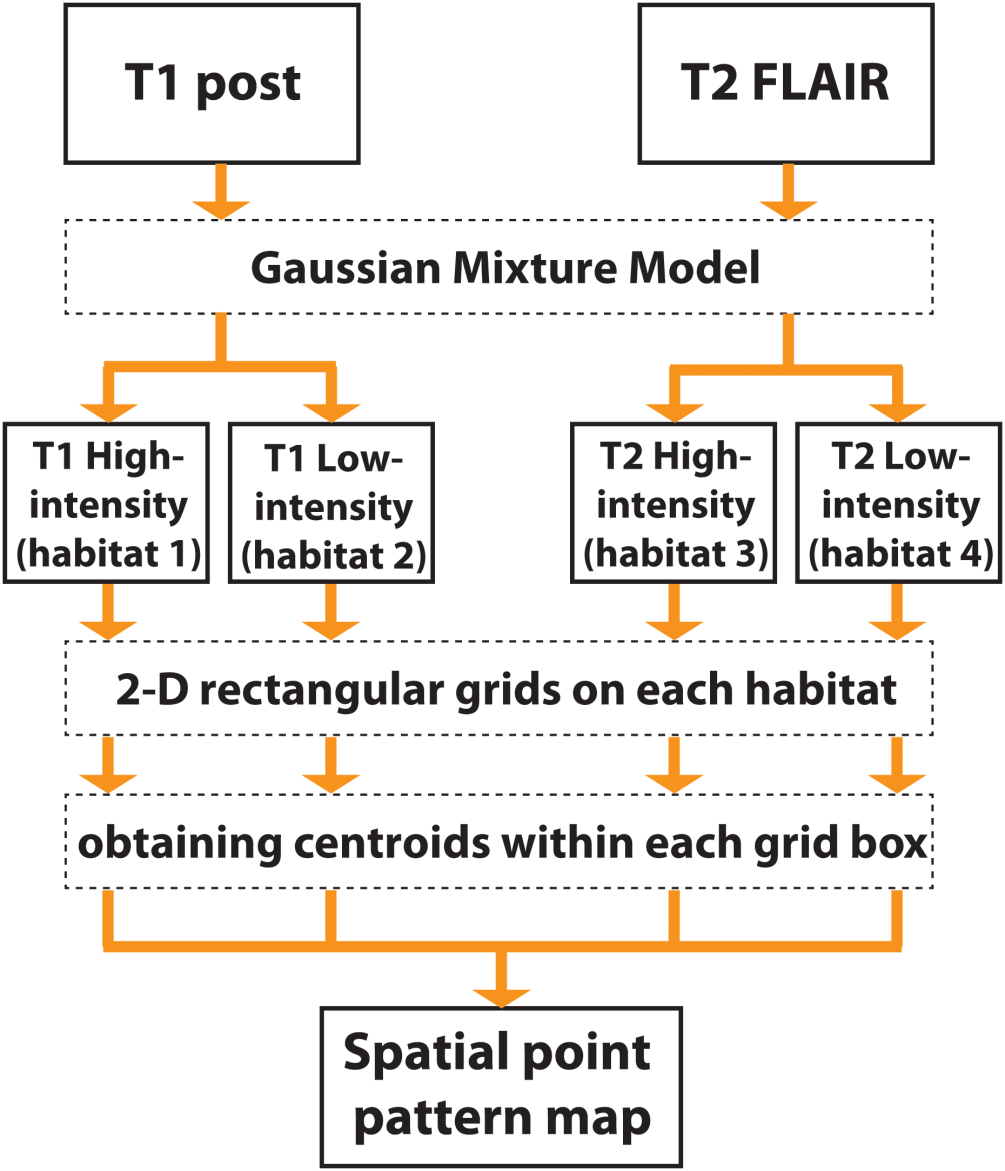
Flowchart. This flowchart represents the global clustering steps for the spatial point pattern map.

Based on the aforementioned clusters identified from the T1 post contrast and FLAIR image data, each pixel (from the tumor ROI of the T1 post-contrast and T2 FLAIR image) is assigned to a low- or high-intensity habitat based on its intensity relative to the threshold. This results in four non-overlapping subpopulations of pixels: a low-intensity T1 habitat, a high-intensity T1 habitat, a low-intensity T2 habitat, and a high-intensity T2 habitat (the T1 and T2 habitats can overlap spatially, however). A spatial point pattern was derived from these habitat regions by overlaying a two-dimensional rectangular grid on the ROIs (Fig 3). Within each grid box, a centroid was estimated from the set of pixels belonging to a specific habitat. The set of all centroids from each of the four habitats, across all grid boxes, specifies a spatial “multitype” point pattern within the tumor region/ROI, *t*(*x*_*i*_) ∈ *T* = {1, …, *S*}, *S* being the set of four habitats, that can then be statistically analyzed [20]. Here, *x*_*i*_ is the co-ordinate of the point and *t*(*x*_*i*_) is its label, ‘type’ or ‘mark’, representing the specific habitat that pixel belongs to. More details are listed in the following section. In Fig 3 the spatial point pattern from an example tumor ROI is indicated as an illustration. Fig 4 shows the flowchart that represents the various processing steps to obtain a spatial point pattern map.

### Feature analysis

Spatial point pattern analysis is a study of the spatial relationships between multiple point patterns. The simplest form of such a point pattern is a point set *X* = {*x*_*i*_: *i* = 1, …} located randomly in **R** ^*d*^ (here, d = 2, since we are looking at a 2D ROI, like in Fig 3). In our study, there are four different “types/marks” (borrowing from terminology in multitype spatial point pattern statistics) of points, *t*(*x*_*i*_) ∈ *T* = {1, …, *S*}, ‘*S*’ representing the four habitats. These represent the T1 low-, T1 high-, T2 low-, and T2 high-intensity habitats, respectively, that can be used to label each point. Thus, the subset of points of type *τ* can be expressed as *X*_*τ*_ = {*x*_*i*_ ∈ *X*_*i*_: *t*(*x*_*i*_) = *τ*}.

For an individual point *x*_*i*_ and point type *τ*, we used the following definition for determining the abundance of neighboring points [21]:

$$\delta (x_{i}):=\sum_{x_{j}\in X\backslash \{x_{i}\}}1(x_{i}\to x_{j}),$$ (1)

$$\delta_{\tau }(x_{i}):=\sum_{x_{j}\in X\backslash \{x_{i}\}}1(x_{i}\to x_{j},t(x_{j})=\tau ),$$ (2)

$$\delta_{=}(x_{i}):=\sum_{x_{j}\in X\backslash \{x_{i}\}}1(x_{i}\to x_{j},t(x_{j})=t(x_{i})),$$ (3)

where *x*_*i*_ → *x*_*j*_ indicates that *x*_*j*_ is a neighbor of *x*_*i*_. *δ*_*τ*_(*x*_*i*_) and *δ*_=_ (*x*_*i*_) represent the abundance of specific types of neighbors and the abundance of the same ‘type’ of neighbors as *x*_*i*_, respectively. 1(.) denotes an indicator variable that is 1 if its argument is true, else it is 0. Based on this framework, we obtained 27 distinct spatial habitat features to characterize the spatial distribution of these four intensity habitats within a tumor.

In addition to the aforementioned features, we derived spatial-association (spatial proximity) features from multitype point pattern analysis such as marked *K*-function, marked *J*-function, marked *G*-function, and inhomogeneous *F*-function [22]. In this study, we used four point “types”, or “marks”, high- and low-intensity T1 post-contrast and high- and low-intensity T2 FLAIR. The marked *G*-function, *G*_*uv*_, also called the “marked nearest neighbor distance function,” estimates the distribution of the distance from a typical point in type *u* to the nearest point of type *v* [23]. Similar to the *G*-function, the *F*-function, *F*_*uv*_, also called the “empty space function” or the “spherical contact distribution,” is defined as probability distribution of the radius of a sphere when it first makes contact with a point in a point process [24]. The marked *K*-function, *K*_*uv*_, also called the “reduced second moment function,” estimates the expected number of points of type *v* within a given distance from a typical point in type *u*. Each of these features quantifies the spatial dependencies (intra-species or inter-species) of these four habitats within the tumor region.

These features were subsequently examined for their association with patient survival rate and GBM molecular subtype status (classical, mesenchymal, neural and proneural). The detailed equations of diversity indices and summary statistics such as *F*, *G*, *J*, and *K* functions for spatial habitat features are described in the supporting information file (S1 Appendix).

### Statistical analysis

Each of the spatial point features described above is a function of spatial distance *r* (either for the definition of the ‘neighborhood’, or explicitly in the definition of the proximity/dependence function) between any two points in a spatial pattern. Thus, we can extract their means and standard deviations as summaries of their variation across *r*. A total of 27 features that consist of the mean and standard deviation of 12 spatial point pattern feature functions (individual species area relationship (ISAR), mingling index, Shannon index, Simpson index, mean composite information (MCI), marked *K* for T1 post-contrast and T2 FLAIR, marked *J* for T1 and T2, marked *G* for T1 and T2, marked *K* for all pairs of the four types of spatial point patterns (corresponding to the four habitats), marked *J* for all pairs, marked *G* for all pairs, and inhomogeneous *F*), and three original spatial point pattern features (ISAR, Shannon index, and Simpson index) were computed from the coordinates of the centroids from the spatial map. For marked point pattern functions, we used both point patterns (T1 post-contrast and T2 FLAIR) individually as well as in pairs for all the four types of spatial point patterns (corresponding to the four distinct habitats). These spatial features (Table 2) were used to predict 12-month overall survival status as well as GBM molecular subtype status (classical, mesenchymal, neural and proneural) using a classification framework. Relationships between spatial habitat features and OS were assessed by Kaplan-Meier and receiver operating characteristic (ROC) analyses [25]. Each feature was dichotomized based on an optimum (cutoff) value derived from ROC analysis. Survival differences between the induced groups were assessed by the K-Adaptive Partitioning (KAP) algorithm [26]. To determine the predictive accuracy of the spatial point pattern features in predicting 12-month survival status and GBM molecular subtype status, a symbolic regression approach [27] was employed, using threefold cross-validation. Specifically, the top 5 features (std-dev *K* multi, mean *K* multi, std-dev *J* multi, mean J multi, std-dev J multi for T1 and T2) based on the coefficient of variation were used to predict both survival status (either > 12 months or ≤12 months) and subtype status. The subtype status is a binary response (one for a specific subtype, zero for other subtypes). The respective classifier’s performance was assessed via ROC curves.

**Table 2:** List of features.

No.	Spatial features	No.	Spatial features	No.	Spatial features
1	ISAR	10	std-dev MCI	19	std-dev J for T1 and T2
2	Mean ISAR	11	Simpson index	20	Mean J
3	std-dev ISAR	12	Mean Simpson index	21	std-dev J
4	Mean mingling	13	std-dev Simpson index	22	Mean G for T1 and T2
5	std-dev mingling	14	Mean K for T1 and T2	23	std-dev G for T1 and T2
6	Shannon index	15	std-dev K for T1 and T2	24	Mean G
7	Mean Shannon index	16	Mean K	25	std-dev G
8	std-dev Shannon index	17	std-dev K	26	Mean F inhomogeneous
9	Mean MCI	18	Mean J for T1 and T2	27	std-dev F inhomogeneous

ISAR, individual-species area relationship; MCI, mean composite information; std-dev, standard deviation.

## Results

**Fig 5:**
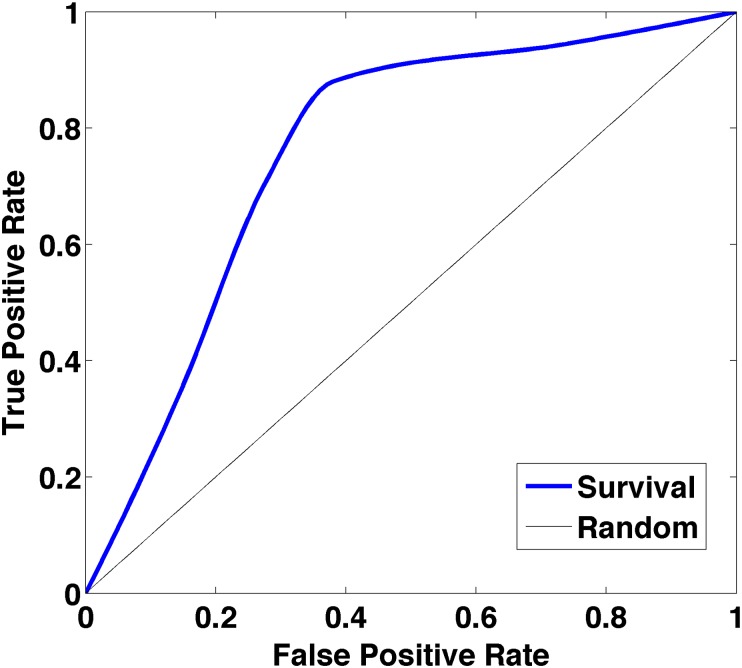
ROC curve for the prediction of survival at the 12-month time point. The x-axis is the false- positive rate, 1 –specificity; the y-axis is the true- positive rate, sensitivity. The area under the ROC curve is 0.76.

Kaplan-Meier survival curves for 14 of the 27 spatial features differed significantly (p < 0.05) at the ROC-derived cutoff (Table 3). The mean ISAR and the standard deviation of the Shannon index had p-values of less than 0.001. For the prediction of OS status based on the top 5 features (based on CoV), the ROC is indicated in Fig 5. The x-axis represents the false-positive rate (FPR or 1 –specificity) and the y-axis represents the true-positive rate (TPR or sensitivity). The optimal threshold was determined by maximizing the sum of sensitivity and specificity. The area under the ROC curve was 0.76. The TPR (sensitivity) and true-negative rate (TNR or specificity) were 0.86 and 0.66, respectively. The accuracy was 0.75 that calculated by using Eq 4 for the optimal model,

$$ACC=\frac{TP+TN}{TP+FN+TN+FP}$$ (4)

where TP, FP, TN, and FN represent true-positive, false-positive, true-negative, and false-negative predictions, respectively. The same procedure was followed to obtain a classifier to predict GBM molecular subtype status using the top five features based on CoV. Accuracy for classical, mesenchymal, neural, and proneural subtypes were 0.88, 0.70, 0.85, and 0.93, respectively, in the ROC analysis for subtype status prediction. TPR, TNR, and accuracy for each subtype were listed in Table 4.

**Table 3:** p-value of survival difference between feature-induced groups (from ROC analysis).

Spatial feature	p-value	Below the ROC cutoff	Above the ROC cutoff
mean ISAR	< 0.001*	52	22
std-dev ISAR	0.003*	46	28
std-dev mingling	0.016*	25	49
Shannon index	0.031	53	21
std-dev Shannon index	< 0.001*	12	62
mean MCI	0.016*	19	55
std-dev MCI	0.003*	52	22
Simpson index	0.031	53	21
std-dev Simpson index	0.004*	22	52
std-dev J for T1 and T2	0.007*	17	57
mean J	0.002*	52	22
std-dev J	0.003*	18	56
mean G	0.023	23	51
std-dev G	0.015*	18	56

ISAR = individual species area relationship; MCI = mean composite information; std-dev = standard deviation.

*These p-values indicate statistical significance even after adjustment for multiple hypothesis testing (using the Benjamini-Hochberg’s FDR-controlling procedure).

**Table 4:** ROC analysis for subtypes (the TPR/TNR/ACC values were obtained at an operating point by maximizing the sum of sensitivity and specificity).

Subtype	TPR	TNR	ACC
Classical	0.578	0.961	0.883
Mesenchymal	0.378	0.917	0.698
Neural	0.373	0.988	0.847
Proneural	0.667	0.984	0.932

## Discussion

In this study, we showed that the spatial features obtained using a multitype spatial point pattern representations of T1 post-contrast and T2-FLAIR MRI data are associated with 12-month survival status and molecular subtypes of GBM. Spatial point pattern analysis with habitat features is widely to assess the abundance, evenness, and spatial interactions of species or point types in a specified area. Some studies have shown that the spatial heterogeneity of CT and MRI intensity values within a tumor is associated with intratumor heterogeneity, tumor malignancy, and patient survival rates [3, 5, 28–30]. In this study, we used the concept of radiologically defined tumor habitats within the tumor based on work by Zhou et al. [3] for clustering pixels based on low- and high-intensity of T1 post-contrast and FLAIR MR images. To our knowledge, this is the first study in which multitype spatial point pattern analysis has been used to obtain spatial features associated with the proximity of intensity habitats within the tumor, and to assess the association of these features measurements with clinical outcomes and molecular subtypes. Therefore, such features can provide a valuable insight into the extent and spatial relationship of tumor sub-regions which could be indicative of tumor heterogeneity. This is an important factor for understanding and characterizing the cause and progression of such tumors. Another key point is that although this study focuses on only two MRI sequences, the concepts and methods presented here are applicable to any number of MRI sequences, thus creating a scalable framework for data that include modalities like T1, T2, FLAIR, dynamic contrast-enhanced and dynamic susceptibility contrast MRI. These methods are also applicable to other disease sites for which multiparametric MRI is used (e.g., prostate, head and neck, etc.).

Our results from the ROC curve show that spatial features from point pattern analysis can discriminate the 12-month survival status of GBM patients with a reasonably high accuracy of 0.75. Also, the corresponding area under the ROC curve value was 0.76, which represents the ability to discriminate between the survival classes. Further, we have high sensitivity (also called the TPR) of 0.86, which represents the high percentage of actual positives that are correctly identified as high survival patients (> 12 months) and have relatively high specificity (also called the TNR) of 0.66, which represents the high percentage of negatives that are correctly identified as low survival patients (≤ 12 months).

This retrospective analysis was performed using a publicly available (TCIA) database, so the scanning protocols and MRI systems are variable across patients; pixel resolutions (256×256 or 512×512), slice thickness (1.4 ~ 5.0mm), repetition time (4.9 ~ 3285.6ms for T1 and 400 ~ 11000ms for T2), and echo time (2.1 ~ 20ms for T1 and 14 ~ 155ms for T2). In this study, we performed intensity scaling as a preprocessing step (based on similar work with TCIA data by Zhou et al., 2014) to minimize the effects of potential variations in MR images from various patients across different scanning or acquisition protocols. However, the effect of these variations in MR images on spatial diversity indices needs to be examined more systematically. Although the level of intensity variation within a tumor has been characterized via tumor habitats, their spatial relationships have not been adequately investigated. The objective of this study is to study the spatial relationship between tumor habitats and assess their relationship with molecular status and clinical outcome. An important direction for follow-up research is the examination of tumor features (cellular morphology, gene expression, metabolism, blood flow, and metastatic potential) that correlate with such spatial features. Such data could enable generation of valuable hypotheses for the investigation of genotype-phenotype relationships based on public domain datasets like TCGA. Further evaluation on larger standardized patient cohorts, along with suitable correction for multiple hypothesis testing, given the number of features examined in this study (twenty seven), is essential to strengthen evidence for the clinical translation of this finding.

In general, the quality of the MR images is very important and key for image analysis. In the study, we investigated the spatial heterogeneity of tumor using the concept of radiologically defined tumor habitats and studied their spatial relationships. Our analysis is based on habitats covered by an 8×8 spatial grid box instead of on a pixel-by-pixel basis, where the grid size was picked empirically in order to trade-off quadrat area and the computational cost of estimating these indices for each grid box. The habitats are defined using a pixel clustering procedure, similar to Zhou et. al., 2014, intended to create only two intensity groups based on pixel intensities (low/high habitats). This process provides some robustness to pixel intensity variations within the image. However, a systematic examination of the nature of index variation with grid size, acquisition/scanning parameters and pixel intensities will be an important consideration for future investigation. Finally, extension of these analyses of spatial diversity indices to 3D tumor regions will also be an important next step.

On the basis of our findings, we conclude that spatial habitat features based on point pattern analysis in MRI-derived tumor habitats may be a promising clinical prognostic tool in radiology studies. Future research should focus on characterizing the biological meaning of these spatial features and exploring a multivariate model such as a multivariate proportional hazards regression analysis incorporating the spatial diversity indices along with clinical variables like age, tumor volume, and Karnofsky performance score to identify combinations of factors with the highest accuracy in predicting disease progression.

## Supporting Information

S1 Appendix Diversity Indicies. Listing of the case IDs for 74 GBM patients (Table A). Top mutated genes in the dataset from eBioPortal (Table B).
(DOCX)
