# Supplementary material for: Spatial Habitat Features Derived from Multiparametric Magnetic Resonance Imaging Data Are Associated with Molecular Subtype and 12-Month Survival Status in Glioblastoma Multiforme
Source: PLoS One. 2015 Sep 14;10(9):e0136557. doi: 10.1371/journal.pone.0136557 (PMC4569439; doi:10.1371/journal.pone.0136557)
Supplement: S1 Appendix — Listing of the case IDs for 74 GBM patients (Table A). Top mutated genes in the dataset from eBioPortal (Table B). (DOCX) [file pone.0136557.s001.docx]

**S1 Appendix - Diversity indices**

In this section, we summarized detailed equations of diversity indices for spatial habitat features such as individual species area relationship (ISAR), spatial mingling index, spatial Shannon index, spatial Simpson index, and mean composite information (MCI). The ISAR, a spatial version of species richness introduced by Wiegand et al. [[31](#_ENREF_31)], quantifies the relationship between an area and the number of individuals of a species within that area. In our study, the term “species” can be considered as different point “types” such as a group of T1 low-, T1 high-, FLAIR low-, and FLAIR high-intensity pixels (habitats). We used the following generalized equation for different types of point neighborhoods:

$\beta_{\tau}(r)=\sum_{\gamma=1}^{S} P_{m,\tau}(\delta_{\gamma}(m)>0)$, (A1)

where $P_{m,\tau}$represents a probability conditioned on a specified event $\tau$ occurring at a typical point *m* $\in X$, and $\delta_{\gamma}(m)$ represents the abundance of neighboring points of type $\gamma$ at point *m*.

The spatial mingling index $M_{\tau}$ is defined as the fraction of point’s *n* nearest neighbors that do not belong to the same type as the reference type $\tau$ [[32](#_ENREF_32)], and a high value of M indicates that many neighbors from different types are located in that point’s vicinity. The mingling index was calculated for a point’s four nearest neighbors (n = 4):

$M_{\tau}(n)=\frac{\bar{\delta}_{\tau,\neq}}{\bar{\delta}_{\tau,=}+\bar{\delta}_{\tau,\neq}}$ (A2)

where $\bar{\delta}_{\tau}$ is the mean local abundances that describe the expected neighborhood composition in the pattern referring to specific type $\tau$.

The Shannon index, an ecological standard measure of diversity, takes into account both the abundance and the evenness of species present in a community where a group of types is presented within an area. A large value of the Shannon index indicates a higher proportion of each species within the community. This index was also generalized to the spatial Shannon index as [[33](#_ENREF_33)]

$H=1-\frac{E(m)}{E(N)}$ (A3)

where *E(N)* is the global entropy and *E*(*m*) is the local entropy that is defined as $E(m)=-\sum_{\tau=1}^{S} \pi_{\tau}\ln\pi_{\tau}$ (A4)

where $\pi_{\tau}$ is the expected frequency of type $\tau$ points in the neighborhood of a typical point *m* of the pattern.

The Simpson index measures the degree of evenness in a community. In this study, we used the local version of the Simpson index that was defined by Shimatani [[34](#_ENREF_34)] and generalized to the spatial Simpson index as [[21](#_ENREF_21)]

$D(r)=1-\sum_{\tau=1}^{S} p_{\tau}\frac{\bar{\delta}_{\tau,\tau}}{\bar{\delta}}$ (A5)

where $\bar{\delta}$ is the mean local abundances describing the expected neighborhood composition in the pattern, $\bar{\delta}_{\tau,\tau}$ is the mean local abundances of neighboring points of the same type, and $p_{\tau}$ is the global counterpart of $\pi_{\tau}$.

The MCI measures a characteristic of the compositional pattern of a group and is defined [[35](#_ENREF_35)] as

$MCI(r)=\sum_{m=1}^{N(r)} -\ln P\left(\frac{F_{m}(r)}{N(r)}\right)$ (A6)

where *F_m_*(*r*) and *N*(*r*) are the number of the combination of point types and the number of points, respectively, within the radius *r* from point *m*, and *P* is the probability of finding different point type combinations.

The multitype *G* function, nearest neighbor distance function, estimates the distribution of the distance from a typical point of type *u* to the nearest point of type *v*:

$G_{uv}(r)=P^{\rho(0,u)}\left\{T_{v}\cap B(0,r)\neq\emptyset\right\}$ (A7)

where $P^{\rho(0,u)}$is the reduced Palm distribution [[23](#_ENREF_23), [36](#_ENREF_36)], *T_v_* is a subset of type *v*, and *B(0,r)* is the closed ball of radius *r* centered at the origin 0.

The multitype *F* function, empty space function, is the distribution function of the distance from an arbitrary fixed point to the nearest point of type *v*:

$F_{v}(r)=P\left\{T_{v}\cap B(0,r)\neq\emptyset\right\}$ (A8)

The multitype *J* function is an index of spatial interaction and defined as

$J_{uv}(r)=\frac{1-G_{uv}(r)}{1-F_{v}(r)}$ (A9)

The multitype *K* function, also referred to as the reduced second moment function, estimates the expected number of points of type *v* within a given distance from a typical point (*x_i_*) in type *u*:

$K_{uv}(r)=E^{\rho(0,r)}\left[\sum_{x_{j}\in T_{v}} 1\left\{x_{j}\in B(x_{i},r)\right\}\right]/\gamma_{v}$ (A10)

where $E^{\rho(0,r)}$is expectation with respect to $P^{\rho(0,r)}$ and $\gamma_{v}$ is the intensity of *T_v_*.

**Table A. Listing of the case IDs for 74 GBM patients**

| **#** | **Case ID** | **#** | **Case ID** | **#** | **Case ID** | **#** | **Case ID** | **#** | **Case ID** |
| --- | --- | --- | --- | --- | --- | --- | --- | --- | --- |
| 1 | TCGA-02-0011 | 16 | TCGA-02-0086 | 31 | TCGA-06-0158 | 46 | TCGA-06-0190 | 61 | TCGA-08-0385 |
| 2 | TCGA-02-0027 | 17 | TCGA-02-0087 | 32 | TCGA-06-0162 | 47 | TCGA-06-0210 | 62 | TCGA-08-0390 |
| 3 | TCGA-02-0033 | 18 | TCGA-02-0102 | 33 | TCGA-06-0164 | 48 | TCGA-06-0213 | 63 | TCGA-08-0392 |
| 4 | TCGA-02-0034 | 19 | TCGA-02-0106 | 34 | TCGA-06-0166 | 49 | TCGA-06-0216 | 64 | TCGA-08-0509 |
| 5 | TCGA-02-0046 | 20 | TCGA-06-0116 | 35 | TCGA-06-0168 | 50 | TCGA-06-0237 | 65 | TCGA-08-0510 |
| 6 | TCGA-02-0047 | 21 | TCGA-06-0122 | 36 | TCGA-06-0171 | 51 | TCGA-06-0238 | 66 | TCGA-08-0511 |
| 7 | TCGA-02-0048 | 22 | TCGA-06-0127 | 37 | TCGA-06-0173 | 52 | TCGA-06-0241 | 67 | TCGA-08-0512 |
| 8 | TCGA-02-0059 | 23 | TCGA-06-0128 | 38 | TCGA-06-0174 | 53 | TCGA-06-0644 | 68 | TCGA-08-0516 |
| 9 | TCGA-02-0060 | 24 | TCGA-06-0129 | 39 | TCGA-06-0175 | 54 | TCGA-06-0645 | 69 | TCGA-08-0518 |
| 10 | TCGA-02-0064 | 25 | TCGA-06-0133 | 40 | TCGA-06-0176 | 55 | TCGA-06-0878 | 70 | TCGA-08-0520 |
| 11 | TCGA-02-0068 | 26 | TCGA-06-0137 | 41 | TCGA-06-0177 | 56 | TCGA-08-0350 | 71 | TCGA-08-0521 |
| 12 | TCGA-02-0069 | 27 | TCGA-06-0145 | 42 | TCGA-06-0179 | 57 | TCGA-08-0353 | 72 | TCGA-08-0522 |
| 13 | TCGA-02-0070 | 28 | TCGA-06-0147 | 43 | TCGA-06-0185 | 58 | TCGA-08-0357 | 73 | TCGA-08-0524 |
| 14 | TCGA-02-0075 | 29 | TCGA-06-0149 | 44 | TCGA-06-0187 | 59 | TCGA-08-0358 | 74 | TCGA-08-0529 |
| 15 | TCGA-02-0085 | 30 | TCGA-06-0154 | 45 | TCGA-06-0189 | 60 | TCGA-08-0360 |  |  |

**Table B. Top mutated genes in the dataset from cBioPortal.** Numbers represent the percentage frequency of those mutation events in the dataset. Only those with frequency > 5% are listed.

| Mutations | EGFR/NF1 | PIK3CA | PTEN | TP53 |
| --- | --- | --- | --- | --- |
| Frequencies (in %) | 5.8 | 5.8 | 11.6 | 13 |
